# Improving Care And Research Electronic Data Trust Antwerp (iCAREdata): a research database of linked data on out-of-hours primary care

**DOI:** 10.1186/s13104-016-2055-x

**Published:** 2016-05-04

**Authors:** Annelies Colliers, Stefaan Bartholomeeusen, Roy Remmen, Samuel Coenen, Barbara Michiels, Hilde Bastiaens, Paul Van Royen, Veronique Verhoeven, Philip Holmgren, Bernard De Ruyck, Hilde Philips

**Affiliations:** Department of Primary and Interdisciplinary Care (ELIZA) – Centre for General Practice (CHA), Faculty of Medicine and Health Sciences, University of Antwerp – Campus Drie Eiken, Universiteitsplein 1, 2610 Antwerp, Wilrijk, Belgium; Department of Epidemiology and Social Medicine (ESOC), Faculty of Medicine and Health Sciences, University of Antwerp – Campus Drie Eiken, Universiteitsplein 1, 2610 Antwerp, Wilrijk, Belgium; Koninklijke Apothekersvereniging van Antwerpen (KAVA), Lange Leemstraat 187, 2018 Antwerp, Belgium

**Keywords:** After hours care, Primary care, Emergency Department, Database, Data linkage, Interdisciplinary

## Abstract

**Background:**

Primary out-of-hours care is developing throughout Europe. High-quality databases with linked data from primary health services can help to improve research and future health services.

**Methods:**

In 2014, a central clinical research database infrastructure was established (iCAREdata: Improving Care And Research Electronic Data Trust Antwerp, www.icaredata.eu) for primary and interdisciplinary health care at the University of Antwerp, linking data from General Practice Cooperatives, Emergency Departments and Pharmacies during out-of-hours care. Medical data are pseudonymised using the services of a Trusted Third Party, which encodes private information about patients and physicians before data is sent to iCAREdata.

**Results:**

iCAREdata provides many new research opportunities in the fields of clinical epidemiology, health care management and quality of care. A key aspect will be to ensure the quality of data registration by all health care providers.

**Conclusions:**

This article describes the establishment of a research database and the possibilities of linking data from different primary out-of-hours care providers, with the potential to help to improve research and the quality of health care services.

## Background

### The changing out-of-hours landscape

The growing need to improve accessibility, quality, efficiency, sustainability and safety of primary care has changed the landscape of out-of-hours (OOH) care in many European countries [1, 2]. The driving forces include: a growing demand for medical care, the changing demographics of the general practitioner (GP) workforce, population growth, ageing and migration, and changes in patient behaviour within a 24/7 culture. More specifically, the increase in average age, feminisation, part-time work and students preferring careers in other medical specialities have had important implications for the GP workforce. Further, the rising costs of health care strain the sustainability of health care systems in many countries [2–4].

Belgian health care is characterised by unlimited access to primary, secondary and tertiary care facilities. Originally, Belgian GPs were continuously available to their patients, and a system of call time rotation was later implemented. During OOH, GPs on duty worked from their own practices, without any professional or administrative support [4]. In 2003, local GPs started up the first General Practitioner Cooperative (GPC) to deliver OOH primary care in Belgium (Deurne-Borgerhout, Antwerp). Currently, the city of Antwerp (the largest city in Flanders, with almost 500,000 inhabitants) is almost fully covered by GPCs. The on-going establishment of GPCs represents one of the most important developments for primary health care in Flanders (Northern part of Belgium, ±6 million Dutch speaking inhabitants). Currently, there are 26 GPCs in Flanders and about 50 % of residents are covered by these GPCs. Typically between 80 and 160 GPs are working in a GPC.

Preliminary figures from our own studies in Flanders show that the introduction of a GPC results in small effects on the workload of GPs and hospitals, but longitudinal data are needed to show any quality changes [5, 6].

During OOH, patients can choose between GP services and Hospital Emergency Department (ED) without former contact or referral. At the ED there is no direct payment as compared to the GP services, where a direct fee for service applies. Medical care is largely reimbursed by medical insurance, which is obligatory in Belgium. Out of pocket payment accounts for approximately 25 % of health expenses. Patients can purchase over the counter medication at pharmacies on call [4].

### Why data collection in OOH?

Research is needed to support the optimisation of patients’ health seeking behaviour as well as the improvement of the organisation and quality of primary OOH care. In surrounding countries, high- quality databases with linked data from primary health services are already successfully used for different research purposes [7, 8]. From the start, Belgian GPCs invested in a high-quality, encoded, electronic registration of patient contacts, i.e. reasons for encounter, clinical diagnosis, and prescriptions. Referrals to other providers (such as EDs), along with core demographic and socio-economic data such as age, gender and reimbursement level are also registered. Originally, these data were collected to allow immediate feedback to the patients’ own GP and to the government, which subsidises the GPCs. These data, however, have the potential to serve other purposes for the benefit of patients, physicians, pharmacists and policy makers. This paper addresses the steps to establish a research database to secure these data for scientific research in Flemish OOH primary care. The data will also inform the future design of health care services.

## Methods

### iCAREdata research database

#### Aim

In 2014, a central clinical research database infrastructure was established (iCAREdata: Improving Care And Research Electronic Data Trust Antwerp, www.icaredata.eu) for primary and interdisciplinary health care at the University of Antwerp (Flanders, Belgium). Currently, this research infrastructure is being developed as a unique, interactive and central clinical research database based on weekly-collected clinical data registered in different OOH primary care settings. To support interdisciplinary collaboration, iCAREdata will collect, link and integrate data from different individual databases unlinked thus far. The aim is to connect and integrate data from all GPCs, EDs of general and academic hospitals and pharmacies involved in OOH primary care in Antwerp (inner city and suburban area). Once established, iCAREdata will provide unprecedented opportunities for OOH primary care research, serve as a state-of-the-art example of clinical data storage and linkage and improve the quality of OOH primary care. Further it will enable follow-up of patients throughout the health care system.

#### Governing the database

iCAREdata (www.icaredata.eu) is coordinated by the Department of Primary and Interdisciplinary Care (ELIZA)—Centre for General Practice (CHA) of the University of Antwerp (www.uantwerpen.be/centre-for-general-practice). The construction and overall safety of the database as well as the coordination of the project is entrusted to a postdoctoral researcher (supervisor—safety and database coordinator). He supervises the quality manager, whose task is to perform the quality control, feedback and research on the database and collaborate with the information and technology (IT) specialist, who is developing the required software tools or searching and adapting existing ones. For the daily management, they collaborate closely with a team of CHA (www.huisartsgeneeskunde.be) researchers dedicated to iCAREdata.

The scientific advisory board is composed of representatives from CHA and all partners involved in the project, i.e. representatives of the four GPCs of Antwerp, EDs of the participating hospitals, professional organisation of pharmacists and a representative of the database funder (Research Foundation Flanders) [9]. The scientific advisory board evaluates the research requests for iCAREdata use on an ad hoc basis.

The steering committee is composed of the supervisor—safety and database coordinator and the senior academic representatives of the research group. Representatives of partners involved are invited on a voluntary basis. The steering committee meets monthly, and oversees the construction, operating and further elaboration of the research infrastructure and resolves management issues.

#### Collected data

Routine clinical data and patient characteristics are collected from the GPCs, EDs and pharmacists electronic records completed during OOH-care on a weekly basis. Unique, individual patient data such as year of birth, gender, postal code, insurability and social security identification code (INSZ code) are registered. For each unique patient contact, the following data are collected during OOH: date and time of arrival, method of contact (by telephone, face to face contact, …), consultation or home-visit, urgency level, identification code of the treating physician (unique code from the National Institute for Health and Disability Insurance (NIHDI)), any referrals or sick leave. Reasons for encounter and diagnosis are registered by the physician using a thesaurus term, and this is mapped to the matching International Classification of Primary Care (ICPC)-2 code. Subjective complaints and clinical examination are captured in free text fields. Finally, drug prescription and matching CNK code (Code Nationale Kode) are collected. This ‘national code’ is mapped to the international Anatomical Therapeutic Chemical (ATC) classification system [10].

At the pharmacy level, the delivered medication, the matching CNK code, the identification code of the prescribing physician (NIHDI code), as well as the date and time of purchase of the medication are collected as ‘medical data’.

In addition to the medical and patient information, iCAREdata will provide valuable information about health care costs. Because of fixed tarrifs used in the Belgium health care system, treatment costs can be derived from the produced data within the OOH care services. This relates to consultations, home visits, medication, diagnostic tests, etc. Thus costs can be calculated indirectly by researchers using the database.

**Fig. 1:**
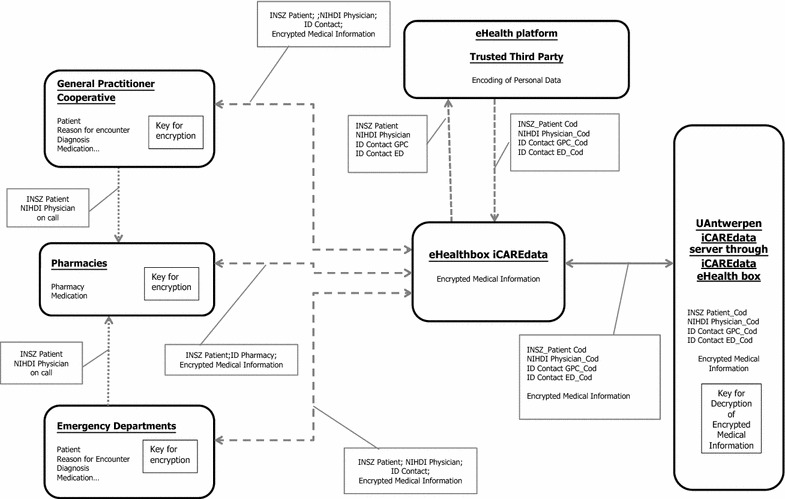
Dataflow of iCAREdata. *ID* identification, *ED* Emergency Department, *GPC* General Practitioner Cooperative, *INSZ* social security number, *NIHDI* personal identification code physician, *_Cod* coded

**Table:** Dataflow: (Fig. 1)

Explanation of used terminology:
Encryption: the process whereby an algorithm is used to reversibly transform data (in this context medical data) for the purpose of covering the real content from interceptors so data can be sent securely from sender to recipient. The algorithm requires one or several keys, depending on the type of encryption used (see further)
Symmetric encryption: a subtype of encryption where sender and recipient use the same key for encryption and decryption
Asymmetric encryption: generally considered a safer subtype of encryption where a key pair is used. The key pair belongs to the recipient and shares one of its keys (i.e. the public key) to authorized senders. This public key is then used for data encryption. The recipient is the sole owner of the other key (i.e. private key) and needs to keep the location of this key secured. The private key can be used to decrypt the encrypted message. The use of keys is interchangeable, and so the choice of public and private key is arbitrary
Encoding: similar to encryption, this process is used to (ir)reversibly transform data. The purpose is to conceal the content from the recipient (see further). This is achieved by first sending the personal data to a Trusted Third Party (TTP). For encoding, only one key is used and the TTP is the sole owner of this key. In case of a reversible encoding process, the same key can be used to decode the data by the TTP
Pseudonymisation: if the same algorithm and key are used by the TTP to encode the same piece of data, the process will always yield the same result. In this context, Pseudonymisation is used to encode personal data (social security number patient, identification number physician) yielding a unique identification cipher, or pseudonym, for these individuals. This way data can be linked to individuals while protecting their privacy by concealing their true identity at the same time.

The records collected from GPCs, pharmacies and EDs include sensitive private medical and personal data from the different actors within primary OOH care (left hand side of Fig. 1). To ensure this data can be transferred to iCAREdata while respecting the privacy of patients and physicians at each step of the data flow, we have chosen to compartmentalise the handling of the medical and personal data (see Fig. 1 and technical details below):

- Medical and demographic data: this data gets encrypted at the source level (GPC, ED, pharmacies). We use the asymmetric encryption services of eHealth (a public governmental institution responsible for the electronic services within the Belgian health care system) [11]. The encrypted data is subsequently sent to a Trusted Third Party (TTP), also eHealth in this context.
- Personal data (INSZ number patient and NIHDI physician code): the TTP encodes this data (centre of Fig. 1). Next, the TTP sends all the data (encrypted medical data and encoded personal data) to iCAREdata’s eHealthBox, another (web-based) service of eHealth. From here, data can be extracted and then imported into iCAREdata.

Upon receiving the encrypted message, iCAREdata decrypts the medical information, keeping the personal data coded (right hand side of Fig. 1). The great advantage of this process is that data from individuals originating from different sources (different GPCs, ED, pharmacies) can be linked to the same individuals, while conforming to privacy regulations. Data is retrieved weekly from iCARAdata’s eHealthBox and imported in the research database, hosted at the University of Antwerp (UA), and stored for 30 years. This enables research on up-to-date information as well as longitudinal trends.

#### Legal and ethical aspects

Ethics approval for data extraction from the electronic medical records for all GPCs in iCAREdata was granted by the Ethics Committee of the University of Antwerp/University Hospital Antwerp (12/49/404 and 13/34/330).

To secure the privacy of information about individual patients, a permission for the data collection at the GPCs was obtained from the Committee of Health of the Commission for the Protection of Privacy (N° 14/094 n173 on November 18th, 2014).(5) A separate application for the data-linkage was approved on July 28th, 2015 (N° 14/194 n133).

Because of its complexity and the knowledge required to query the database, and especially because the database information is still vulnerable even after thorough encryption, each potential investigator has to make an official request to the scientific advisory board and can only apply when permission of a licensed ethical committee has already been acquired. The scientific advisory board reviews the final purpose and scientific goal of each research request. In general, this procedure is necessary for all research questions and feedback to the applicant should not take more than 3 weeks. If no objections are formulated, the request is handed over to the safety and database coordinator to perform the appropriate queries. Direct interaction between the requester and the database coordinator is recommended at this stage.

Publication of research results making use of iCAREdata is encouraged whatever the findings, without interference from the original data owners. However, publications should always cite the iCAREdata and the original databases involved.

The supervisor—safety and database coordinator and the quality controller have direct access to the iCAREdata server. They have to report their activities regarding iCAREdata to the scientific advisory board. For this purpose, a provenance tool monitors and visualizes all actions with the database.

### Collected data use by different interest groups

UA, CHA is expected to be the prime, but not the only, user of iCAREdata. Research projects of UA, CHA have to follow the general procedure as described below.

GPC managers and managers of other databases (EDs, pharmacies) involved in the project have the possibility to consult their own data after pseudonymisation according the directives of eHealth and the Commission for the Protection of Privacy. They can perform their own queries on their own data without interference from other partners. When they wish to study data from other providers, they have to make an official request to the scientific advisory board.

Other researchers (universities, public health institutions, scientific organisations, private *industry and companies*) not involved in the project have to apply for access to the scientific advisory board if they have research questions needing one or more of the databases included in iCAREdata. Because of privacy issues relating to individual patients or so called “small cells”, only the data manager coordinator has access to data at the individual patient level. When access is needed on individual patient level, permission from the Committee of Health of the Privacy Commission has to be obtained by the requesting party.

No direct access is allowed for individual healthcare workers, patients or the public. Healthcare workers and patients from whom data were collected will be granted permission to consult and to ask corrections of their own personal information. An opt-out procedure is available.

At this phase of the project we have not yet addressed the issue of fees and payments for researchers using the database. This will become an issue for decision within the Steering Committee and Scientific Advisory Board when data are ready to be used by external researchers.

### Research and quality improvement opportunities of the iCAREdata database

iCAREdata provides new opportunities for research and supporting quality of care. To be able to accomplish this, obtaining qualitative data is a key issue for this project.

By training the GPs from participating GPCs on how to improve registration of clinical information, the quality of registered data will be enhanced. CHA offers specific training to implement ICPC2-coded registration of clinical data, more specifically: reason for encounter (RFE) and diagnosis. Participating GPCs are offered feedback reports on their data quality. This intervention should lead to quality improvement in data registration [12]. Also offering Thesaurus terms and ‘favourite lists’ of the most frequent RFEs and diagnosis, improves the quality of ICPC2-coding by physicians at the GPC [13].

In addition to improving quality of registration, the data can also be used for clinical epidemiological research, e.g. on the diagnosis and management of a number of conditions. The initial focus would be on infectious diseases (e.g. respiratory and urinary infections). Further, the impact of interventions to improve the management of these conditions can be assessed as we did in former research [14–16]. Finally, iCAREdata would allow complementing the evaluation of the impact of public health campaigns and the implementation of guidelines with respect to infectious disease management and to other topics. Impact of national and European antibiotic awareness campaigns such as BAPCOC and GRACE, TRACE can be evaluated, using the iCAREdata database [17].

iCAREdata information has multiple purposes, including feedback reports for individual GPCs or EDs, benchmarking, and advising different stakeholders such as the Ministry of Health. Feedback on numerous items can be provided to health care professionals and their management and can serve as a way of benchmarking their performance (qualitative and quantitative). Annual reports, required by the government, can be easily produced. The database also facilitates easy access to aggregated data, benchmarking and surveillance. It will be possible to measure the quality of care provided by GPs using indicators that quantify quality of care [14]. For instance, improving antibiotic prescription in Belgium needs attention, both the percentage of patients receiving an antibiotic as well as prescribing the guideline recommended antibiotic. Relevant indicators are the share of quinolones for simple cystitis among females or antimicrobial prescription for acute cough.

Data will also allow documentation of the whole chain of care because it can provide an overview on: the characteristics of the population contacting the GPC, the ED and/or pharmacist on call, the incidence of the reasons for encounter and diagnoses, the work burden for all personnel (e.g. identification of periods of high patient turnover, etc. allowing better organisation of work force), the influence of new services on patient choices, patient preferences in OOH care, the effect of co-payment and out-of-pocket payment on patient behaviour, and the influence of triage systems on patient flows. This offers the opportunity to optimise the quality, safety, and the organisation of OOH care.

## Discussion

Until now, individual patient data from different services were not linked, nor could groups of patients be followed throughout the health care system. iCAREdata will allow us to study the quality of care more profoundly with a special focus on health benefits.

In the EurOOHnet network, we collaborate with EU partners on data sharing [2]. The use of data of iCAREdata supports more comparative research at the regional, national, and European level.

### Added value and challenges of the iCAREdata database

#### Strengths

This project is the first of its kind in Belgium to securely link and integrate data from OOH care from GPCs, EDs and pharmacies, thereby allowing exploration of a broad range of research questions about the health care system. Our research group has broad experience with data linkage, for example as an active partner in the TRANSFoRm project [18].

iCAREdata complements a project, started in 1990 by the Department of General Practice of the Katholieke Universiteit Leuven, on an integrated computerized network (Intego; www.intego.be) to provide information on incidence and prevalence of diseases, laboratory tests and treatments in Flanders (Belgium). Although very valuable data on primary care are gathered in that project, the clinical data registered by Intego do not capture OOH primary care, do not allow linkage, lack information on the reason for encounter and contain data from less than 100 GPs [19]. Because all GPs in Belgium are obliged to participate in OOH care services, the iCAREdatabase project, offers a novel platform to collect data from all 400 GPs in this region during OOH care.

By using an automated procedure of data transfer, there is no extra burden for participating GPs in iCAREdata. Since computerisation gives inexpensive and easy access to large volumes of data, researchers will be able to retrospectively analyse a large amount of valid data, adhering the most recent ethical and privacy rules. In addition, the implementation of quality enhancing projects in OOH care can be analysed with iCAREdata. Another potential added value is providing GPCs with information that can act as catalyst for behaviour change during office hours. Linking OOH data with data from during office hours is the logical next and future step.

#### Weaknesses

Quality of data depends on practitioners’ registration performance. There is an enormous variability in the quality of data extracted from GPs’ electronic medical records [20]. The electronic medical record uses a predetermined field to enter information on the reason for encounter and diagnosis using a thesaurus term, which is then automatically converted to ICPC-2 code. Individual practitioners can choose to ignore this list and fill in their own free text, making it far more complicated to run queries on this data. Learning to register the reasons for encounter and diagnoses with appropriate thesaurus terms [21], is a crucial part of training offered by the iCAREdata project. The training aims to substantially improve quality of registration during OOH as well as during office hours.

Another issues is that practitioners on call base codes on a correct diagnosis or assessment. Criteria based diagnoses are more accurate than symptom based diagnoses, but experience with registration is lacking among Belgian physicians [19]. Capturing uncertainty about the final diagnosis is another issue to that merits further consideration, because these items will determine the value of electronic-health record-based data. The crucial issue in our project is how to best optimise the data registration [20].

## Conclusions

Routinely collected primary care computer data aggregated into large databases can be used for auditing, quality improvement, health service planning and clinical epidemiological research [22]. By integrating the data of different actors in OOH care and linking data from individual patients, the database will become attractive for research and health services planning. Careful and cautious handling of this information is necessary and possible. The establishment of iCAREdata provides unprecedented opportunities of OOH care research, and serves as a state-of-the-art example of clinical data storage, integration and improve the quality of out-of-hours care. The latter includes the promise of a spillover effect in daily care.

## Abbreviations

ATC: anatomical therapeutic chemical classification system
CHA: Centrum voor Huisartsgeneeskunde Antwerpen, Department of General Practice Antwerp
CNK: Code Nationale Kode
ED: Emergency Department
GP: General Practitioner
GPC: General Practitioner Cooperative
ICPC: International Classification of Primary Care
INSZ: insurability and social security identification code
NIHDI: National Institute for Health and Disability Insurance
OOH: out of hours
TTP: Trusted Third Party
RFE: reason for encounter
UA: University of Antwerp

## References

[1] Huibers L (2009). Out-of-hours care in western countries: assessment of different organizational models. BMC Health Serv Res.

[2] Huibers L (2014). EurOOHnet-the European research network for out-of-hours primary health care. Eur J Gen Pract.

[3] http://data.worldbank.org/indicator/SH.XPD.TOTL.ZS/countries/xj?display=graph.

[4] Philips H. Out-of-hours care in Belgium. Dissertation for the degree of doctor in Medical Science at the University of Antwerp, 2010. ISBN: 9789057283109.

[5] Philips H (2010). What’s the effect of the implementation of general practitioner cooperatives on caseload? Prospective intervention study on primary and secondary care. BMC Health Serv Res.

[6] Wens J (2005). Use of emergency departments by primary care patients. Eur J Gen Pract.

[7] Hippisley-Cox J, Stables D, Pringle M (2004). QRESEARCH: a new general practice database for research. Inform Prim Care.

[8] http://www.nivel.nl/en/dossier/nivel-primary-care-database.

[9] http://www.fwo.be/en/.

[10] WHO, WHO Collaborating Centre for Drug Statistics Methodology. Anatomical therapeutic chemical (ATC) classification system: guidelines for ATC classification and DDD assignment 2010. Oslo; 2011.

[11] https://www.ehealth.fgov.be/nl/support/basisdiensten/systeem-voor-end-end-vercijfering.

[12] Jordan K, Porcheret M, Croft P (2004). Quality of morbidity coding in general practice computerized medical records: a systematic review. Fam Pract.

[13] Ryckebosch P (2013). Hoe kan gecodeerd registreren makkelijker gemaakt worden?. Huisarts Nu.

[14] Adriaensens N, et al. Quality of antibiotic prescription during office hours and out-of-hours in Flemish primary care, using European quality indicators. Eur J Gen Pract. 2013. Early online. 10.3109/13814788.2013.828200 23998298

[15] Willems L (2012). Can we improve adherence to guidelines for the treatment of lower urinary tract infection? A simple, multifaceted intervention in out-of-hours services. J Antimicrob Chemother.

[16] Philips H (2014). Guidelines adherence to lower urinary tract infection treatment in out-of-hours primary care in European countries. Qual Prim Care.

[17] Philips H (2013). Use of out-of-hours services: the patient’s point of view on co-payment a mixed methods approach. Acta Clin Belg.

[18] Okkes IM (2002). The March 2002 update of the electronic version of ICPC-2—a step forward to the use of ICD-10 as a nomenclature and a terminology for ICPC-2. Fam Pract.

[19] Truyers C (2014). The Intego database: background, methods and basic results of a Flemish general practice-based continuous morbidity registration project. BMC Med Inform Decis Mak.

[20] de Clercq E (2012). Quality assessment of automatically extracted data from GPs’ EPR. Stud Health Technol Inform.

[21] Okkes I, et al. The March 2002 update of the electronic version of ICPC-2: a step forward to the use of ICD-10 as a nomenclature and a terminology for ICPC-2. Fam Pract. 2002; 19(5):543–6. www.rivm.nl/who-fic/ICPC-2.htm. Accessed on 14 Feb 2014. 10.1093/fampra/19.5.543 12356710

[22] de Lusignan S, van Weel C (2006). The use of routinely collected computer data for research in primary care: opportunities and challenges. Fam Pract.

